# Visualization and analysis of mapping knowledge domains for optic neuritis: a bibliometric research from 2013 to 2022

**DOI:** 10.1007/s10792-024-02948-7

**Published:** 2024-02-12

**Authors:** Bo Jiang, Nan Hong, Fangkun Zhao, Feng Dong

**Affiliations:** 1 Department of Ophthalmology, The First Affiliated Hospital, Zhejiang University School of Medicine, Hangzhou, 310003 China; 2 Department of Ophthalmology, The Fourth Affiliated Hospital of China Medical University, Shenyang, 110032 China

**Keywords:** Optic neuritis, Multiple sclerosis, Neuromyelitis optic, VOSviewer, CiteSpace, Bibliometric analysis

## Abstract

**Purpose:**

To explore the global research trends, hotspots and frontiers of optic neuritis (ON) over the past decade through qualitative and quantitative analysis of bibliometrics.

**Methods:**

Publications on ON from 2013 to 2022 were retrieved from Web of Science Core Collection (WoSCC). VOSviewer and CiteSpace were mainly used to facilitate bibliometric analysis and visualization.

**Results:**

A total of 3027 papers were retrieved from peer-reviewed publications and the annual research output increased over time. Neurosciences neurology was the most published area. The USA was the most productive and influential country, and in the focus of international cooperation. University College London was the most productive organization and Charite Medical University of Berlin had the largest number of cooperating partners. Paul F contributed the largest number of publications and Wingerchuk DM ranked first among the co-cited authors. *Multiple Sclerosis and Related Disorders* was the most prolific journal publishing ON research. The most co-cited references mainly focused on the diagnostic criteria for neuromyelitis optica spectrum disorder (NMOSD) and multiple sclerosis (MS). The keywords formed the following four clusters: the pathophysiology of MS-ON; the autoantibody markers and diagnostic criteria of NMOSD-ON and myelin oligodendrocyte glycoprotein associated disorder-ON (MOGAD-ON); the epidemiology and clinical characteristics of ON; and the treatment of ON.

**Conclusion:**

This bibliometrics analysis showed a systematic view of the evolutionary process, research hotspots, and future directions of ON research. It can provide insights for ON research and valuable information for neuro-ophthalmologic specialists to evaluate research policies and promote international cooperation.

**Supplementary Information:**

The online version contains supplementary material available at 10.1007/s10792-024-02948-7.

## Introduction

Optic neuritis (ON) is an inflammation of the optic nerve with *a* variety of etiologies. It is a blinding optic nerve disease worldwide that occurs most frequently in young and middle-aged individuals. The annual incidence of ON is 0.56–5.36 per 100 000 population with a higher incidence in females [1, 2]. The latest classification divides ON into autoimmune (usually relapsing), and infectious (including post-infectious or post-vaccination) or systemic (with systemic disorders). According to consensus opinion, autoimmune ON includes aquaporin 4 antibody associated ON (AQP4-ON), myelin oligodendrocyte glycoprotein antibody associated ON (MOG-ON), multiple sclerosis associated ON (MS-ON), collapsin response mediator protein 5 associated ON (CRMP5-ON), single isolated ON (SION), relapsing isolated ON (RION), and chronic relapsing inflammatory ON (CRION) [3]. The treatment and prognosis of ON depend on the underlying causes. In recent decades, with the continuous progress in ON research, substantial developments have occurred in epidemiology, pathophysiology, neuroimaging, diagnostic work up and treatment, and numerous classic articles have been produced, as well as a number of review articles on different aspects of ON [1, 2, 4–6]. Therefore, there is a need to comprehensively collate the vast and complex literature on the subject in order to visualize the intellectual framework of the research field in a holistic manner.

Bibliometric analysis is a widely used qualitative and quantitative method to explore the knowledge structure and development of specific research fields using mathematical and statistical methods. The search results using the Web of Science (WoS) database were exported to software platforms such as VOSviewer and CiteSpace for further analysis [7, 8]. There have been a few studies addressing the scientometric analysis in ophthalmology research, such as keratoconus, ocular graft-versus-host disease and ocular drug delivery [7, 9, 10]. In the present study, we represented the first bibliometric analysis of ON research over the past decade (2013–2022). This study provided a comprehensive analysis of the current status, global hotspots and trends in the field to guide future research direction, which allows researchers or doctors to easily access information on the disciplines, and serves as a useful reference for this topic.

## Methods

### Data sources and search strategy

**Fig. 1:**
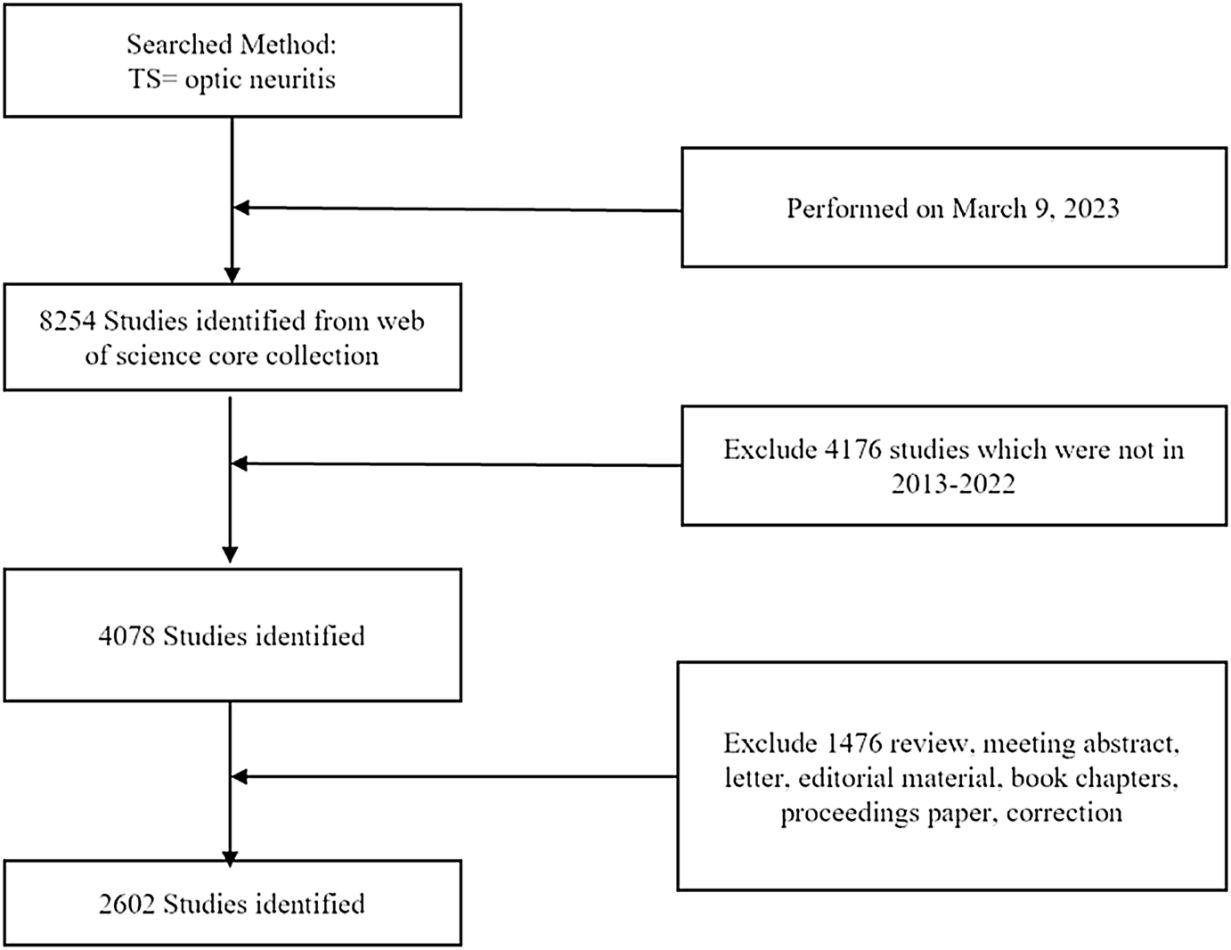
Flowchart of Data collection

In the present study, the literature from the Science Citation Index Expanded database of the WoS Core Collection (WoSCC) was retrieved on a single day (March 15 2023). The retrieval strategy is shown in Fig. 1 The topic was ‘‘optic neuritis,’’ the publication period was from “2013 to 2022,” the document type was ‘‘article and review,’’ and the language was “English.” The retrieved results were exported as ‘‘tab delimited file’’ with ‘‘full record and cited references.’’ Data from each publication included title, author, journal, year, country, organization, keywords, abstract, citation references, and other relevant information.

### Analytical tools and statistical methods

After that, data were uploaded into Microsoft Excel 2019, VOSviewer and CiteSpace software for bibliometric and visual analysis. VOSviewer (http://www.vosviewer.com) is a computer program for constructing and visualizing bibliometric maps [11]. In this study, data were imported into VOSviewer (v.1.6.16) and systematically analyzed. The most common bibliometric techniques mainly include co-authorship, co-citation and co-occurrence analysis. Co-authorship analysis reveals patterns of collaboration among countries, organizations, and authors. Co-citation analysis, which examines articles or authors that tend to be cited together, can indicate a strong conceptual relationship between the studies. Co-occurrence analysis reveals how often two words appear together in the same article to identify how close they are, thereby demonstrating hot topics and trends in the discipline [7, 8]. In the knowledge maps generated by VOSviewer, different nodes represent different research elements. Relationships between elements are presented by links between nodes. The colors of nodes and links indicate different clusters. Cluster analysis was used to summarize research hotspots and frontiers in this research field. The thickness and length of links between nodes represent the strength of the connection and relevance between corresponding nodes.

CiteSpace (version 6.2.R3) was used to construct dual-map overlay of journals, detect references and keywords with citation burst, and create timeline view of keywords. The dual-map overlay of journals allows the identification of patterns showing how specific domains (citing journals) are influenced by other domains (cited journals), and the colored paths represent the citation relationships, indicates the citation trajectory and knowledge flow [12]. Citation burst in references or keywords refer to documents or keywords frequently cited by articles during a period, which can illustrate the evolution of the knowledge domain [13].

## Results

### Overview of publications

**Fig. 2:**
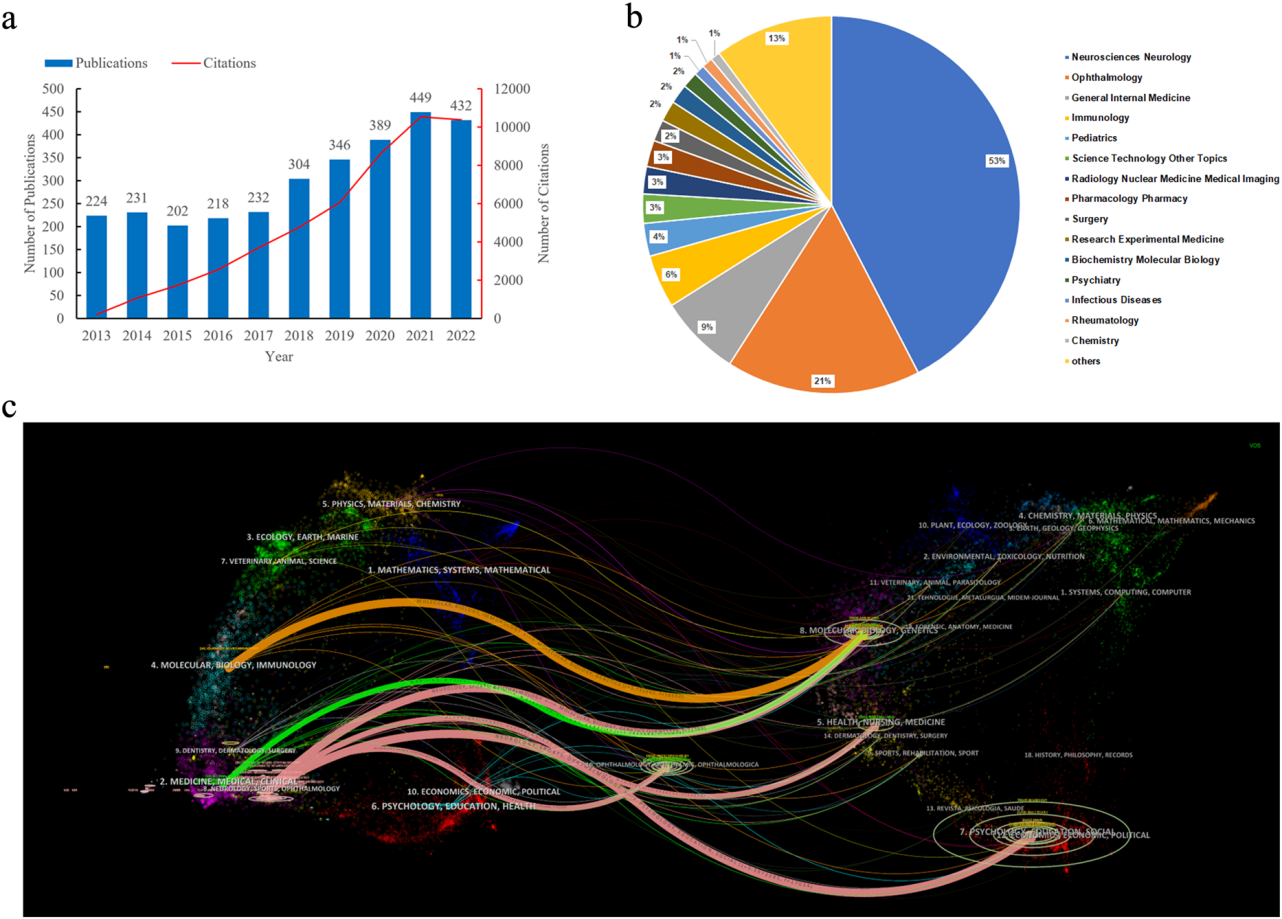
Overview of publications. **a** The annual number of publications and citations in ON research from 2013 to 2022. **b** Research areas of publications. **c** Dual-map overlay of journals

A total of 3027 documents were identified by our search strategy (Fig. 1). Figure 2a shows a gradually increasing trend in the number of published articles and total number of citations during the last decade, although it fluctuated slightly. Publications exceeded 300 in 2018 and 400 in 2021.

The publications on ON could be concentrated on 72 areas according to the research areas in the Web of Science (WoS) database. Figure 2b shows that neurosciences neurology (53%) was the most published area, followed by ophthalmology (21%), general internal medicine (9%), immunology (6%) and pediatrics (4%).

Figure 2c shows a dual-map overlay of journals. The left side (citing journals) of the pink, orange and green curves represented the published articles which were mainly in the field of neurology, ophthalmology, medicine, clinical, molecular, biology and immunology, whereas the right side (cited journals) of the curves represented the sources of references cited which were mainly focused on psychology, education, health, medicine, molecular, biology and genetics.

### Distribution and co-authorship of productive countries

**Fig. 3:**
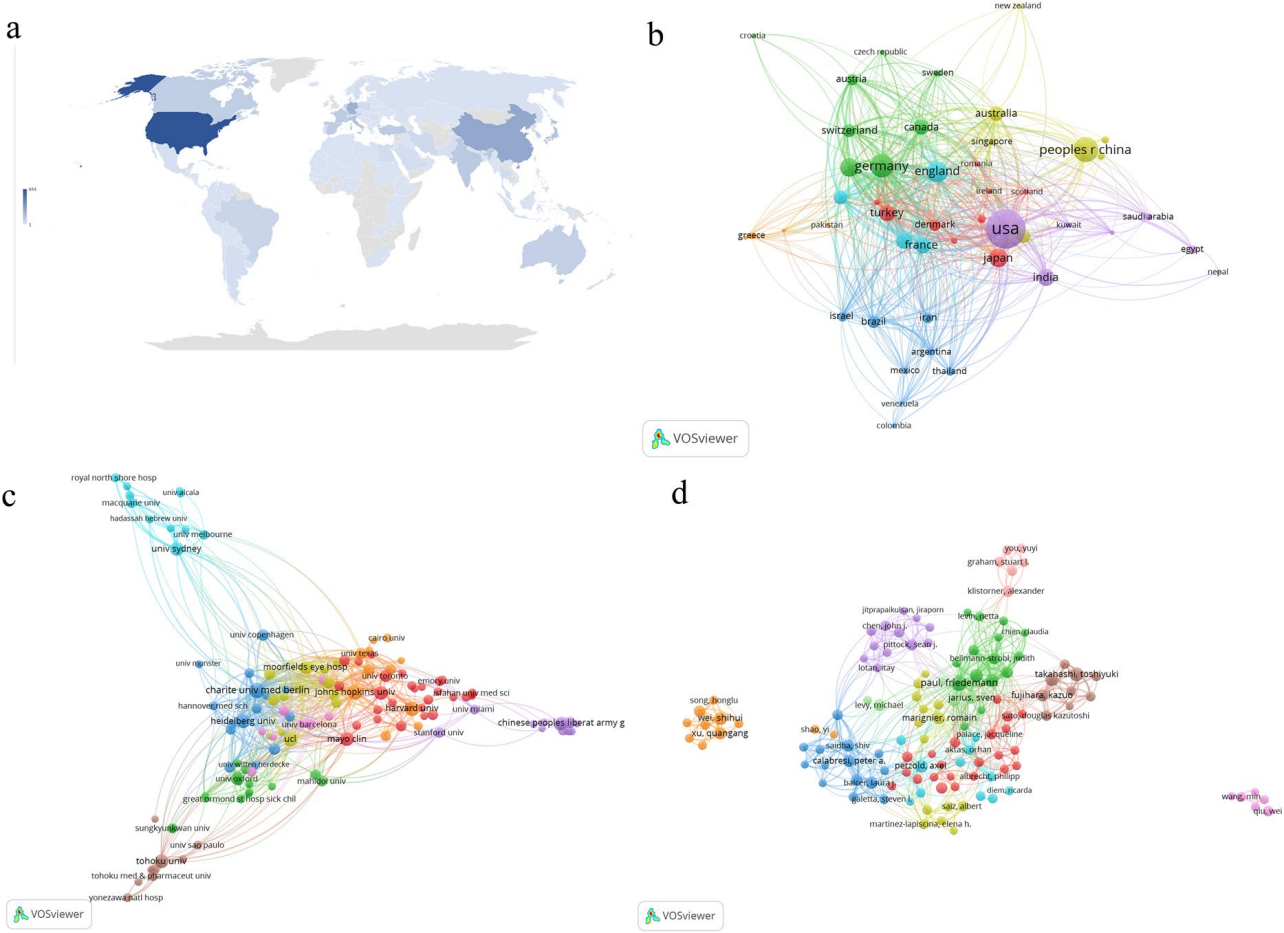
Distribution of the documents and Bibliometric analysis of the co-authorship. **a** Distribution of countries/regions contributed to research. The color shade reflects the number of literature. **b** The co-authorship network of productive countries. **c** The co-authorship network of research organizations. **d** The co-authorship network of productive authors. In co-authorship analysis, the node size indicates the number of publications

Figure 3a shows the world distribution of the number of documents published by countries or regions. Based on the retrieved results, the 3027 articles originated from 118 countries. According to Table 1, the top 10 countries published 2671 articles, accounting for 88.24% of the total. The USA contributed the most publications (844, 27.88%), followed by China (349, 11.53%), Germany (290, 9.58%), England (245, 8.09%) and Japan (192, 6.34%). The citation analysis showed that the USA ranked first with 16,863 total citations, followed by Germany (10,507) and England (10,026), but the England had the largest number of citations per paper (40.92). Figure 3b depicts the cooperation between countries/regions. When the minimum number of documents of a country was set to 10, 47 countries reached the threshold. The USA had the largest number of cooperating partners (45), followed by Germany (42), England (41), Italy (38) and France (38).

**Table 1:** Top 10 productive countries, organizations and main source journals in ON research from 2013 to 2022

Rank	Subject	Count (%)	Total Citation	Citation per paper
	*Country*			
1	USA	844 (27.88)	16,863	19.98
2	China	349 (11.53)	3014	8.64
3	Germany	290 (9.58)	10,507	36.23
4	England	245 (8.09)	10,026	40.92
5	Japan	192 (6.34)	4824	25.13
6	Italy	184 (6.08)	5080	27.61
7	India	155 (5.12)	1107	7.14
8	Turkey	146 (4.82)	1175	8.05
9	France	140 (4.63)	4743	33.88
10	Spain	138 (4.56)	5295	38.37
	*Organization*			
1	University College London (England)	120 (3.96)	5524	45.65
2	Harvard University (USA)	104 (3.44)	1681	16.16
3	Charite Medical University of Berlin (Germany)	102 (3.37)	5533	54.25
4	Johns Hopkins University (USA)	99 (3.27)	3314	33.82
5	University of Pennsylvania (USA)	76 (2.51)	3210	42.24
6	Mayo Clinic (USA)	74 (2.45)	3488	47.78
7	University of Sydney (Australia)	69 (2.28)	2891	41.9
8	Tohoku University (Japan)	66 (2.18)	2915	44.17
9	Heidelberg University (Germany)	65 (2.15)	3457	53.18
10	Moorfields Eye Hospital (England)	59 (1.95)	2097	34.95
	*Journal*			
1	Multiple Sclerosis and Related Disorders	178 (5.88)	1755	9.86
2	Multiple Sclerosis Journal	114 (3.77)	4066	35.67
3	Journal of Neuro-Ophthalmology	81 (2.68)	726	8.96
4	Frontiers in neurology	71 (2.35)	1797	25.31
5	Journal of Neurology	71 (2.35)	811	11.42
6	PLoS One	63 (2.08)	1176	18.67
7	Journal of Neuroimmunology	54(1.78)	768	14.22
8	Journal of the Neurological Sciences	52 (1.72)	3325	63.94
9	Neurology	52 (1.72)	587	11.29
10	Neuro-Ophthalmology	37 (1.22)	110	2.97

^a^Percentages (%) were calculated by dividing the row count by the total number of publications (*n* = 3027)

### Distribution and co-authorship of research organizations

Based on the retrieved results, 3027 articles were published by 3505 organizations. The top 10 organizations published 834 articles, accounting for 27.55% of the total. The most productive research organizations were University College London (120, 3.96%, 5524 citation), Harvard University (104, 3.44%, 1681 citation), and Charite Medical University of Berlin (102, 3.37%, 5533 citation) (Table 1).

Figure 3c depicts the cooperation between organizations. When the minimum number of documents in an organization was set to 10, 127 reached the threshold. The co-author network contained 125 organizations and was divided into nine clusters represented by different colors. The largest cluster (red), consisting of 29 organizations, centered on University of Pennsylvania, Mayo Clinic and University of Toronto. Charite Medical University of Berlin had the largest number of cooperating partners (*n* = 72), followed by Johns Hopkins University (*n* = 71), and University College London (*n* = 63).

### Distribution of source journals

Retrieved articles were published in 681 journals. Table 1 lists the top 10 journals on this topic. The highest number of articles (178, 5.88%) were published in Multiple Sclerosis and Related Disorders, followed by Multiple Sclerosis Journal (114, 3.77%) and Journal of Neuro-Ophthalmology (81, 2.68%). The top 10 journals have totally published 773 publications, accounting for 25.54% of the total articles.

### Distribution, co-authorship and co-citation of authors

A total of 13,384 authors have contributed to the research on ON. Among all authors, Paul F from Charite Medical University of Berlin (92, 5307 citations) ranked first, followed by Brandt AU from Charite Medical University of Berlin (56, 2865 citations) and Pelzold A from University College London (52, 2004 citations). The top 10 authors have published 505 articles, accounting for 16.68% of the total (Table 2).

**Table 2:** Top 10 productive authors and co-cited authors in ON research from 2013 to 2022

Rank	Author	Count	Citation	Citation per paper	Country	Co-cited author	Co-citation	Country
1	Paul F	92	5307	57.68	Germany	Wingerchuk DM	1362	USA
2	Brandt AU	56	2865	51.16	Germany	Jarius S	1151	Germany
3	Pelzold A	52	2004	37.81	England	Petzold A	548	England
4	Takahashi T	48	1497	31.19	Japan	Polman CH	532	Netherlands
5	Wei SH	48	406	8.46	China	Beck RW	514	USA
6	Jarius S	44	3151	71.61	Germany	Lennon VA	454	USA
7	Calabresi PA	43	1981	46.07	USA	Costello F	374	Canada
8	Nakashima I	43	2078	48.33	Japan	Saidha S	353	USA
9	Marignier R	41	1818	44.34	France	Kitley J	342	England
10	Fujihara K	38	2861	75.29	Japan	Ramanathan S	327	Australia

Author co-authorship analysis provides valuable information for individual researchers seeking collaboration opportunities. When the minimum number of documents from an author was set to 10, 132 reached the threshold. The co-authorship network of authors, including 125 authors, was divided into eleven clusters represented by different colors. The largest cluster (red) consisting of 22 authors centered on Frederiksen KL from University of Copenhagen. The number of collaborators with Paul F was 63, followed by Brandt AU (*n* = 40) and Petzold A (*n* = 40) (Fig. 3d).

Author co-citation analysis is used to determine the academic researchers who have played an important role in this field. Wingerchuk DM from Mayo Clinic (1362 co-citations) ranked first, followed by Jarius S from University of Heidelberg (1151 co-citations) and Petzold A from University College London (548 co-citations) (Table 2).

### Citation and co-citation of references

A Total of 3027 articles were cited 51,091 times, with an average of 16.88 citation per paper. Table 3 lists the top 10 most cited articles on ON. “Distinction between MOG antibody-positive and AQP4 antibody-positive NMO spectrum disorders,” published in Neurology in 2014, was the most cited articles (548 citations) [14].

**Table 3:** Top 10 cited references in ON research from 2013 to 2022

Rank	Cited references	Title	Citations
1	Sato DK, Neurology. 2014;82(6):474–81	Distinction between MOG antibody-positive and AQP4 antibody-positive NMO spectrum disorders	548
2	Filippi M, Lancet Neurol. 2016 Mar;15(3):292–303	MRI criteria for the diagnosis of multiple sclerosis: MAGNIMS consensus guidelines. (review)	480
3	Jarius S, J Neuroinflammation. 2016;13(1):279	MOG-IgG in NMO and related disorders: a multicenter study of 50 patients. Part 1: Frequency, syndrome specificity, influence of disease activity, long-term course, association with AQP4-IgG, and origin	400
4	Kitley J, JAMA Neurol. 2014;71(3):276–83	Neuromyelitis optica spectrum disorders with aquaporin-4 and myelin-oligodendrocyte glycoprotein antibodies: a comparative study	395
5	Jarius S, J Neuroinflammation. 2016;13(1):280	MOG-IgG in NMO and related disorders: a multicenter study of 50 patients. Part 2: Epidemiology, clinical presentation, radiological and laboratory features, treatment responses, and long-term outcome	357
6	Tintore M, Brain. 2015;138(Pt 7):1863–74	Defining high, medium and low impact prognostic factors for developing multiple sclerosis	317
7	Toosy AT, Lancet Neurol. 2014;13(1):83–99	Optic neuritis (review)	315
8	Jurynczyk M, Brain. 2017;140(12):3128–3138	Clinical presentation and prognosis in MOG-antibody disease: a UK study	312
9	Ramanathan S, J Neurol Neurosurg Psychiatry. 2018;89(2):127–137	Clinical course, therapeutic responses and outcomes in relapsing MOG antibody-associated demyelination	309
10	Petzold A, Lancet Neurol. 2017;16(10):797–812	Retinal layer segmentation in multiple sclerosis: a systematic review and meta-analysis (review)	285

**Fig. 4:**
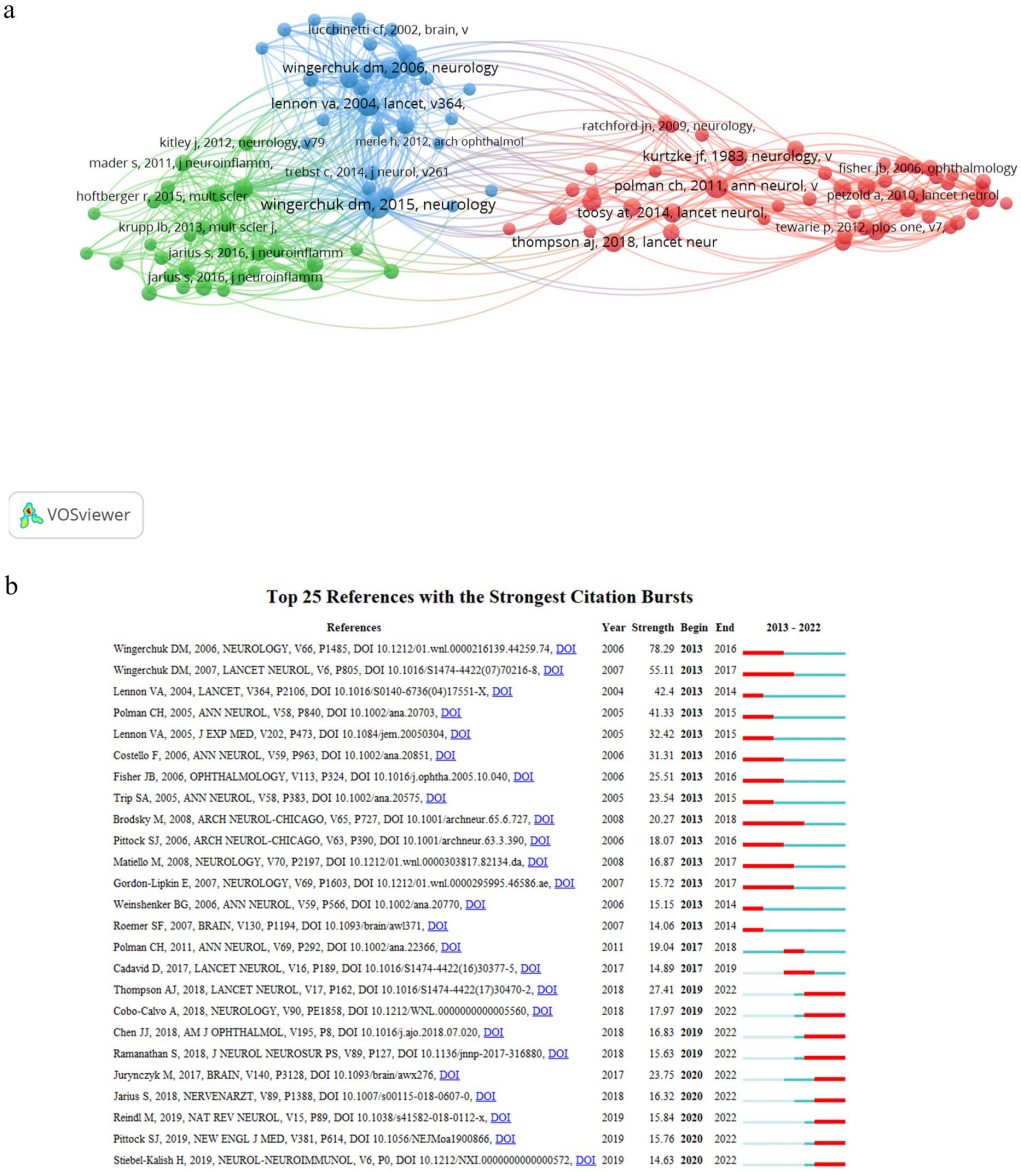
**a** Co-citation network of reference. The node size represents the counts of co-citations. **b** The top 25 references with the highest burst value. The red bars mean references cited frequently, and the green bars were references cited infrequently

Reference co-citation analysis is essential for identifying and analyzing research evolution and trends in a given field. The top 10 co-cited references are presented in Table 4. Publications with the title “International consensus diagnostic criteria for neuromyelitis optica spectrum disorders” ranked first by Wingerchuk DM in 2015 [15]. The minimum citation number of a cited reference was set to 60. Among the 54,220 co-cited references, 110 references reached the threshold. Figure 4a shows the three main clustering results of references co-citation analysis. The remaining references appearing in Fig. 4a are listed in Supplement 1.

**Table 4:** Top 10 co-cited references in ON research from 2013 to 2022

Rank	Co-cited Reference	Title	Cluster	Citations
1	Wingerchuk DM, Neurology.2015;85:177	International consensus diagnostic criteria for neuromyelitis optica spectrum disorders	3	617
2	Polman CH, Ann Neurol. 2011;69:292	Diagnostic criteria for multiple sclerosis: 2010 Revisions to the McDonald criteria	1	418
3	Lennon VA, Lancet. 2004;364:2106–12	A serum autoantibody marker of neuromyelitis optica: distinction from multiple sclerosis	3	382
4	Wingerchuk DM, Neurology.2006;66:1485	Revised diagnostic criteria for neuromyelitis optica	3	339
5	Wingerchuk DM, Lancet Neurol. 2007;6:805	The spectrum of neuromyelitis optica	3	293
6	Beck RW, N Engl J Med. 1992;326:581	A Randomized, Controlled Trial of Corticosteroids in the Treatment of Acute Optic Neuritis. The Optic Neuritis Study Group	1	231
7	Sato DK, Neurology. 2014;82(6):474–81	Distinction between MOG antibody-positive and AQP4 antibody-positive NMO spectrum disorders	2	230
8	Thompson AJ, Lancet Neurol. 2018;17(2):162–173	Diagnosis of multiple sclerosis: 2017 revisions of the McDonald criteria	1	225
9	Kurtzke JF, Neurology. 1983;33:1444	Rating neurologic impairment in multiple sclerosis An expanded disability status scale (EDSS)	1	221
10	Wingerchuk DM, Neurology. 1999;22;53(5):1107–14	The clinical course of neuromyelitis optica (Devic's syndrome)	3	221

Figure 4b lists the top 25 references with the strongest citation between 2013 and 2022. The reference with the strongest citation burst (78.29) was “Revised diagnostic criteria for neuromyelitis optica” by Wingerchuk DM in 2006 [16] with burst duration from 2013 to 2016. Notably, eight of the 25 references were still in burstness. Among them, one reference related to the diagnostic of MS [17], five references related to MOGAD, one related to eculizumab treatment in AQP4-NMOSD [18], and the other one highlighted the importance of early treatment for the long-term visual recovery in ON with AQP4 and MOG immunoglobulin (IgG) [19].

### Co-occurrence of keywords and burst keywords

**Fig. 5:**
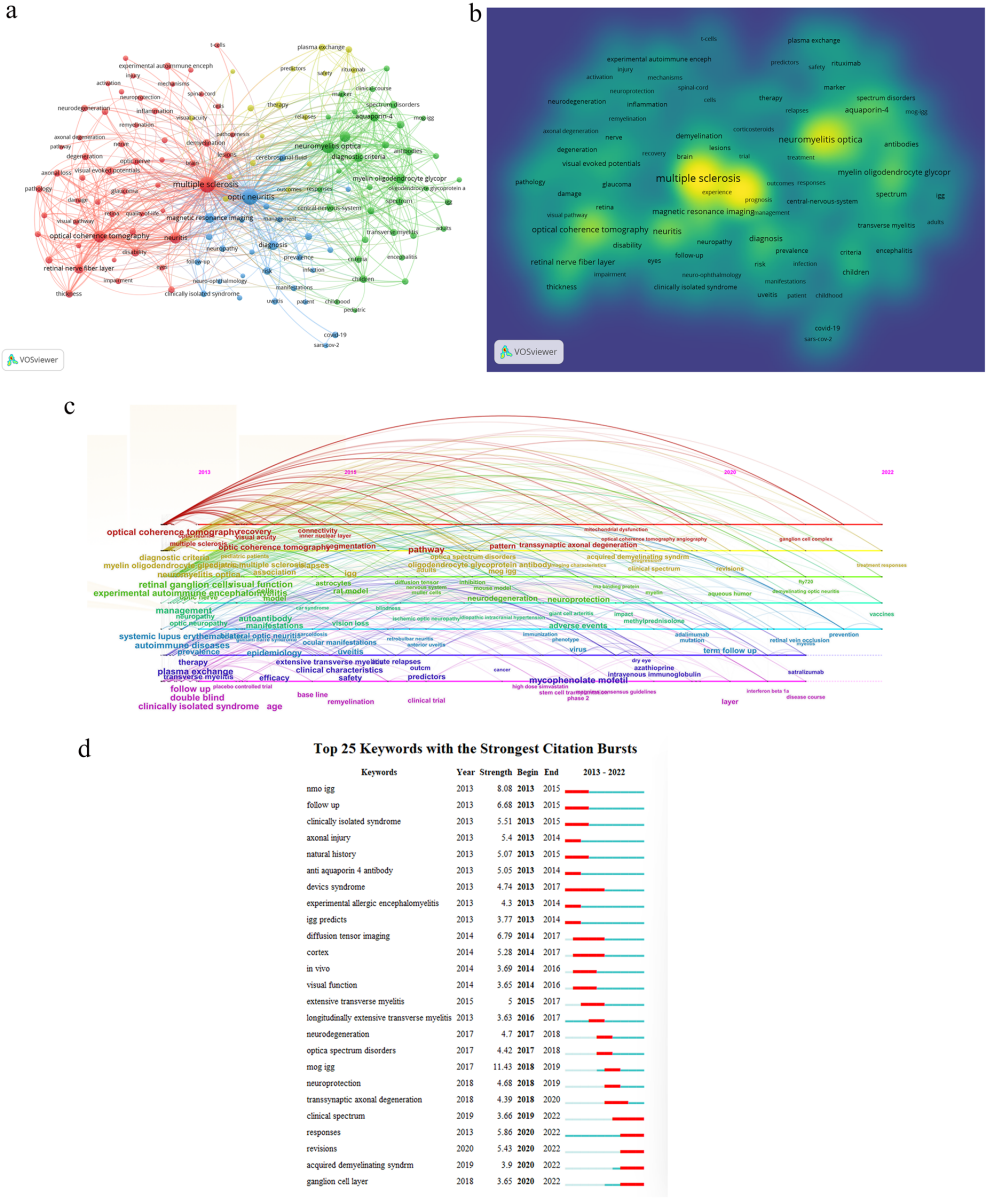
Co-occurrence of Keywords and burst keywords in ON studies. **a** Network visualization of keywords co-occurrence. The node size indicates the frequency of occurrence. **b** Density visualization of keywords co-occurrence. **c** Timeline view of keywords. **d** The top 25 keywords with the highest burst value

Using co-occurrence analysis, developing trends and hot topics of ON research were identified. The minimum number of occurrences of a keyword was set to 30 (1% of all publications). Among the 7477 keywords that were associated with ON research, 130 keywords reached the threshold. MS, ON, neuromyelitis optica (NMO), optical coherence tomography (OCT), neuritis, NMOSD, MOG, magnetic resonance imaging (MRI), AQP4 and diagnostic criteria were high-frequency keywords. The top 10 keywords for each cluster are listed in Table 5. Figure 5a shows the clustering of keywords based on this network, and four clusters are represented. In the density visualization of keywords from VOSviewer, the same identified keywords mapped by frequency of appearance, and keywords in yellow occurred with the highest frequency (Fig. 5b).

**Table 5:** Co-occurrence analysis of keywords (Top 10 keywords in the 4 clusters)

Cluster 1	Cluster 2	Cluster 3	Cluster 4
Multiple sclerosis (1339)	Neuromyelitis optica (681)	Optic neuritis (1272)	Therapy (91)
Optical coherence tomography (480)	Neuromyelitis optica spectrum disorder (329)	Magnetic resonance imaging (280)	Plasma exchange (88)
Neuritis (397)	Myelin oligodendrocyte glycoprotein (281)	Diagnosis (228)	Double-blind (78)
Retinal nerve fiber layer (236)	Aquaporin-4 (256)	Risk (122)	Efficacy (75)
Disability (153)	Diagnostic criteria (239)	Clinical-features (93)	Rituximab (63)
Visual evoked potentials (147)	Antibodies (174)	Cerebrospinal-fluid (89)	Trial (58)
Demyelination (131)	Spectrum (152)	Optic neuropathy (82)	Relapses (57)
Thickness (124)	Children (141)	Clinically isolated syndrome (81)	Experience (50)
Axonal loss (106)	Multicenter (117)	Neuropathy (80)	Safety (45)
Pathology (99)	Central-nervous-system (113)	Prevalence (71)	Outcomes (44)

^a^The numbers in brackets indicate the frequency of occurrence

The research frontier of ON can be further visualized and analyzed with the Timeline view from CiteSpace (Fig. 5c). In 2013–2015, the main keywords were MS, OCT, MOG, NMO, retinal ganglion cells, experimental autoimmune encephalomyelitis, autoimmune diseases, plasma exchange (PE) and clinical isolated syndrome. In 2015–2020, the main keywords were optical coherence tomography angiography (OCTA), transsynaptic axonal degeneration, MOG-IgG, neurodegeneration, neuroprotection, adverse events, mycophenolate mofetil (MMF) and so on. In 2020–2022, the main keywords were ganglion cell complex, fty720, vaccines, prevention, satralizumab, interferon beta-1a and so on.

Figure 5d lists the top 25 keywords with the strongest citation between 2013 and 2022. The keyword with the strongest citation burst was MOG-IgG most recent bursts are responses, revisions, acquired demyelinating syndrome and ganglion cell layer (GCL).

## Discussion

### Characteristics of the papers

This study provides a bibliometric analysis on global trends, hotspots and frontiers of ON research during 2013–2022. Our bibliometric analysis showed a steady increase in ON academic publications over the 10-year period. The change in the number of articles indicated that this field of study was receiving more attention from both academic and clinic communities, reflecting the gradual prosperity and stable development trend. Neurosciences neurology, ophthalmology and general internal medicine were the most prominent research area, account for 83% of the total.

The dual-map overlay of journals reveals the overall scientific contributions [12]. We found that neurology, ophthalmology, medicine, clinical, molecular, biology and immunology were the significant application fields, and psychology, education, health, medicine, molecular, biology and genetics were the research foundation. This suggested that the research field of ON mainly focused on neurology and ophthalmology, with a broad spectrum of basic research ranging from non-medical, such as psychosocial and education to clinical and molecular biology.

### Global contribution to ON research

Analysis of the most productive countries showed the top 10 countries included six in Europe, one in North America and three in Asia. Co-authorship analysis indicated that the USA was an international scientific center for ON research, and cooperation between countries had played a vital role in the progress and development of ON research.

Among the top 10 organizations, five were from the USA, three from Europe, one from Asia, and one from Australia. The distribution of research organizations was consistent with the distribution of countries. Significant cooperation was observed between organizations. Notably, the cooperation relationships among organizations are not restricted by geographic distance. In terms of the number of links, Charite Medical University of Berlin, Johns Hopkins University, University College London, University of California San Francisco and University of Barcelona had the highest numbers, indicating that these research organizations were at the core of the entire research network.

The top 10 active journals included seven neurology journals, two neuro-ophthalmology journals and one multidisciplinary journal. This indicates the interdisciplinary and complex nature of ON. It is worth noting that these journals are mainly in the field of neurology, while the number of articles in the field of ophthalmology is relatively low, reflecting the lack of attention to this field by ophthalmologists and the need to strengthen the relevant research. Journal of the Neurological Sciences (JCR Q2) had the highest average citation rate (63.94), reflecting the high academic impact of this journal.

The top 10 most cited papers were published in the speciality journals, and 7 of them are original papers. More than half of them (6/10) involved the clinical characteristics of MOG-IgG disease or comparative studies of MOG-IgG versus AQP4-IgG disease. Others involved MRI criteria and prognostic factors for MS, and retinal layer segmentation in MS.

Based on references co-citation analysis, high-quality publications have provided a useful theoretical and practical foundation for the field of ON. As shown in Fig. 2d, the co-cited references were divided into three clusters. Cluster 1 (red) consisted mainly of the investigation into ON generally associated with MS or those at a risk of conversion to MS-ON, including diagnostic criteria, assessment of axonal loss and visual dysfunction, and treatment. As shown in Table 4, four of the top 10 co-cited references focused on this field. A publication with the title “Diagnostic criteria for multiple sclerosis: 2010 Revisions to the McDonald criteria” by Polman CH ranked second [20]. These criteria for the diagnosis of MS are widely used in research and clinical practice, with the most recent being the 2017 McDonald criteria [17]. Cluster 2 (green) consisted mainly of the investigation into NMOSD and MOGAD, including diagnostic criteria, multicenter studies, and comparative studies between AQP4-IgG and MOG-IgG diseases. Publications with the title “Distinction between MOG antibody-positive and AQP4 antibody-positive NMO spectrum disorders” ranked seventh [14]. In 2012, MOG-IgG was reported in a proportion of AQP4-IgG-seronegative NMOSD patients [21, 22]. Therefore, the majority of studies in this cluster were published from 2012 onwards. Cluster 3 (blue) mainly included studies of NMO prior to 2012, providing a historical view of the origins of NMO. Publications with the title “International consensus diagnostic criteria for neuromyelitis optica spectrum disorders” ranked first by Wingerchuk DM [15], and “A serum autoantibody marker of neuromyelitis optica: distinction from multiple sclerosis” ranked third by Lennon VA [23]. Furthermore, the authors of the top co-cited references generally correspond to the top co-cited authors.

### Analysis of the hot topics and trends

Due to the increased number of publications during this decade, it is impossible to read them all. Therefore, constructing a network diagram of research hotspots can help track internal relationships of the literature and help researchers deepen the understanding of scientific findings in research hotspots. Based on keyword co-occurrence analysis, as shown in Fig. 5a, the topics of ON mainly formed four clusters.

Cluster 1 (red) represents keywords associated with the pathophysiology of MS-ON. The most frequently extracted keywords included “MS,” “OCT,” “neuritis,” “retinal nerve fiber layer (RNFL),” “disability,” “visual evoked potentials (VEP),” “demyelination,” “thickness,” “axonal loss,” “lesions” and “pathology.”

Typically, MS is an autoimmune neurodegenerative disease characterized by inflammation, demyelination, and axons loss in the central nervous system (CNS) [4]. Both genetic (e.g., HLA DRB1*15:01) and environmental (e.g., vitamin D and cigarette smoking) factors contribute to MS susceptibility. Immunological factors, with their effector cells (B and T lymphocytes, activated macrophages and microglia), are known to be important in the pathogenesis of MS. Visual symptoms are common and ON is the onset symptom of MS in 25% of cases and occurs in approximately 70% of patients during the disease [1].

Within this cluster, OCT was the second most frequent term co-occurring with ON (*n* = 480). During the past two decades, OCT has evolved and matured into an interesting and highly sensitive structural retinal imaging biomarker for early recognition and monitoring of inflammation, axonal and neural degeneration in MS [24]. OCT has contributed to greater insights into the pathophysiology of MS. The macular ganglion cell layer and inner plexiform layer (mGCIPL) and peripapillary RNFL (pRNFL) showed thinning in MS-ON eyes (− 16·42 μm and − 20.10 μm) and in MS without ON (MS-NON) eyes (− 6·31 μm and − 7.41 μm) compared with control eyes [24]. An important advantage of measuring mGCIPL compared to pRNFL is the earlier detection of atrophy. Thinning of mGCIPL becomes quantifiable at 1 month after MS-ON, while the recommendation for pRNFL is to wait at least 3 months. Furthermore, analysis of the mGCIPL remains useful in cases of severe atrophy of the pRNFL following MS-ON, whereas the floor effect may prevent observation of further atrophy around the optic disk [6, 24]. In MS-NON, the possibility of mGCIPL and pRNFL thinning could be caused by previous subclinical ON episodes or primary degeneration of the mGCIPL neurons due to MS. Another mechanism is axonotmesis in the visual pathway is thought to cause retrograde transsynaptic axonal degeneration, which will inevitably lead to atrophy of the inner retinal layers’ atrophy [25]. Although there was no difference in the thickness of the combined outer plexiform layer and outer nuclear layer in MS-ON or MS-NON eyes compared with control eyes, the inner nuclear layer (INL) thickness is rendered as an attractive marker of inflammatory activity, and thickening of this layer (0.77 μm) was more substantial in MS-ON eyes than in MS-NON eyes [6, 24]. Inter-eye differences (IEDs) of 5 μm for pRNFL (sensitivity 71%, specificity 65%) and 4 μm for GCIPL (sensitivity 68%, specificity 77%) are useful diagnostic criteria for asymptomatic and symptomatic unilateral MS-ON from healthy controls [26]. Moreover, this standard has recently been included in the paraclinical criteria of ON [3]. Studies also indicate that the pattern of mGCIPL and pRNFL loss could potentially help differentiate between MS and some differential diagnoses or MS subtypes [27, 28]. The presence of NMO-ON is associated with more pronounced thinning of pRNFL and GCL, and more frequent microcystic macular edema than in MS. Furthermore, subclinical changes in pRNFL are less common in NMO than in MS [27]. Previous study found lower pRNFL thickness and mGCIPL volume correlate with more severe brain and spinal cord atrophy, and appear more closely associated with disability than MRI volumetric measures [29]. Significant correlations were also found between OCT parameters and VEP latency, neurodegenerative biomarkers, visual function and outcome for neuroprotective or disease-modifying therapies [30–33].

OCTA is a new technology developed in 2016 that may facilitate studies of neurovascular coupling in MS. Studies employing OCTA have shown that retinal vascular plexus densities are reduced in MS and particularly within the superficial vascular plexus (SVP) which mainly supplies the GCL [32, 34]. IEDs in SVP density and mGCIPL thickness correlate well with visual function in MS-ON patients, and detectable changes in SVP density after ON may occur after changes in mGCIPL thickness [32].

Cluster 2 (green) represents keywords associated with the autoantibody markers and diagnostic criteria of NMOSD-ON and MOGAD-ON. The most frequently extracted keywords included “NMO,” “NMOSD,” “MOG,” “AQP4,” “diagnostic criteria,” “antibodies,” “spectrum,” “children,” “multicenter” and “CNS.”

NMO, also known as Devic disease, is a severe inflammatory CNS disorder distinct from MS. Prior NMO diagnostic criteria required a history of ON and myelitis. The most pivotal advance in 2004 was Lennon’s discovery that a serum IgG autoantibody (NMO-IgG) was a specific marker for NMO and then they showed that NMO-IgG binds selectively to the AQP4 water channel located in astrocytic foot processes [23, 35]. In 2015, the International Panel for NMO Diagnosis has developed a new nomenclature defines the unifying term NMOSD (divided into NMOSD with or without AQP4-IgG), which included ON, acute myelitis, area postrema syndrome, acute brainstem syndrome, acute diencephalic clinical syndrome, and symptomatic cerebral syndrome [15]. ON (42%) is the second most common initial manifestation of NMOSD, and 63% of patients with NMOSD ultimately develop ON. Presumedly, 73–90% of NMOSD patients have AQP4-IgG [5]. Previous studies have shown that coexisting autoimmune disorders are present in more than one third of AQP4-IgG patients (e.g., systemic lupus erythematosus or Sjögren syndrome) [15, 36]. More recently another antigenic target, MOG has been identified, accounting for approximately 40% of NMOSD patients who are seronegative for AQP4 [37]. MOG is expressed on the surface of CNS myelin and oligodendrocytes and is a target for autoimmune responses that results in CNS inflammation and demyelination [38]. MOGAD is related to a diverse spectrum of clinical manifestations, including ON (mostly recurrent), myelitis, brainstem encephalitis and acute disseminating encephalomyelitis (ADEM)-like presentations [37]. In addition, whether a fraction of seronegative NMOSD patients may have other disease-specific antibodies remains undetermined.

Cluster 3 (blue) represents keywords associated with the epidemiology and clinical characteristics of ON. The most frequently extracted keywords included “ON,” “MRI,” “diagnosis,” “risk,” “clinical-features,” “cerebrospinal-fluid (CSF),” “optic neuropathy,” “clinically isolated syndrome,” “neuropathy” and “prevalence.” Herein, the discussion mainly focuses on MS-ON, AQP4-ON and MOG-ON.

The proportion of AQP4-ON (USA and Europe: 0–5.8%, China: 20–43.5%, South Korea:14.3–37.8%, Japan: 3.4–12.4%) or MOG-ON (USA and Europe: 1.7–13.8%, China: 14.4–21.8%, Japan: 10.2–25.7%) among all ON cases appears to be much higher in Asian populations than in white Caucasians from Europe and North America, and appears to correlate with a lower prevalence of MS in these regions [1, 2, 39]. Further epidemiological studies of ON in other countries, particularly in Africa, Latin America, and the Middle East, are needed to formulate a more global picture.

Much of our understanding of typical ON comes from the Optic Neuritis Treatment Trial (ONTT). The classic clinical presentation of typical idiopathic-ON or MS-ON is subacute monocular visual loss associated with pain during eye movement in young adults, developing over hours or days. The degree of vision loss varies widely and tends to reach its nadir within 2 weeks. Fundoscopy reveals optic disk edema in one-third of cases. Visual recovery usually begins within the first few weeks, and prognosis for visual acuity is good with more than 90% of patients returning to 20/40 or better [1, 5]. While typical ON is a clinical diagnosis, the risk stratification for the future development of MS can be assessed by the number of cerebral white matter lesions on the baseline MRI [40]. The 2017 McDonald criteria for MS allow a diagnosis of MS if CSF-specific oligoclonal bands are associated with a typical clinically isolated syndrome (a patient’s first “attack”) and clinical or MRI demonstration of dissemination in space [4].

There are important differences including clinical characteristics and prognosis of AQP4-ON and MOGA-ON [56]. Overall, the median age of onset of NMOSD is around 40 years, approximately 10 years later than that of MS and MOGAD, and MOGAD is more common in children than in adults. The most common manifestation of MOGAD is ADEM in children < 7 years, and ON or transverse myelitis in older children and adults [5, 41]. There is a striking female predominance (female to male ratio 9:1) for NMOSD, whereas it appears to be no obvious gender bias in the incidence of MOGAD [39]. Bilateral involvement is similar to that seen in AQP4-ON (~ 50%) and MOG-ON, but 3–4 times greater than in MS-ON. MOG-ON is commonly (76–86%) associated with optic disk edema, which can be severe enough with peripapillary hemorrhages, with optic disk edema noted in a minority of cases of AQP4-ON (5–33%) and typical ON (9.5–35%) [5]. ON can potentially lead to initial severe vision loss. The results have demonstrated that visual prognosis after the first episode of AQP4-ON is worse than that of typical ON and MOG-ON. AQP4-ON is marked by relapses often associated with poor recovery and long-term visual disability [5, 42]. Patients with MOG-ON often present with AQP4 phenotype or CRION. Patients with MOG-ON have favorable visual outcomes despite recurrent ON attacks (50%): final visual acuity ≤ 20/200 in at least one eye in 5–20% of patients, compared to at least 50% in AQP4-ON. Seronegative conversion or lowering titers are associated with monophasic disease course in MOG-ON, and CSF testing for MOG-IgG is useful in seronegative cases for diagnostic purposes [43]. Compared with MS-ON patients (showing enhancement of short segment), MRI with contrast shows longitudinally extensive enhancement of more than half of the prechiasmic optic nerve length in 80% of MOG-ON cases, and perineural enhancement in 50% of cases. In contrast, AQP4-ON commonly involves the posterior optic nerve/chiasmal. As in AQP4-ON, the CSF findings in MOG-ON are not diagnostically confirmatory [5, 41]. Identifying new potential biomarkers may help clinicians to manage of patients with ON. Cytokines (especially IL-6) may be useful to distinguish NMOSD/MOGAD from MS and may be a short-term prognostic biomarker [43]. Glial fibrillary acidic protein (GFAP) and neurofilament light chain (NfL) are both candidate blood biomarkers for diagnosing and monitoring NMOSD and MS [43, 44].

COVID-19, which emerged at the end of 2019, is also contained in this cluster (Fig. 5a). In literature, new onset or recurrence of ON has been reported after COVID-19 infection and vaccination. [45–47]. It is recommended that patients who are predisposed or already diagnosed with autoimmune or autoinflammatory disorders should be carefully assessed the benefits and risks of COVID-19 vaccination.

Cluster 4 (yellow) represents keywords associated with the treatment of ON. The most frequently extracted keywords included “therapy,” “PE,” “double-blind,” “efficacy,” “rituximab,” “trial,” “relapses,” “experience,” “safety” and “visual acuity.”

Treatment standards of acute typical ON with intravenous methylprednisolone (IVMP) can speed up visual recovery but do not improve final visual outcomes [40]. However, a study from Canada found that bioequivalent doses of oral corticosteroids (1250 mg/day) for 3 days could be used as an alternative to intravenous corticosteroids (1 g/day) for 3 days [48]. Delayed treatment initiation for vision loss may lead to poor prognosis in all kinds of ON, with a critical time interval of 48 h [19, 49]. Several disease-modifying treatments included interferon beta-1b, interferon beta-1a, fingolimod (fty720) and Glatiramer acetate have been discovered and approved for patients with relapsing–remitting MS and clinically isolated syndrome who have positive demyelinating lesions at brain MRI [4].

Acute treatment with IVMP is the first-line therapy for AQP4-ON and MOG-ON patients. PE targets specific antibodies, complement and several proinflammatory proteins, and is generally considered as a rescue treatment for ON patients with poor response to corticosteroid, especially in AQP4-ON [50, 51]. Studies demonstrated improved Extended Disability Status Scale (EDSS) outcome in NMO relapses receiving the PE combined with IVMP when compared with IVMP alone, especially in patients taking preventive immunosuppressive medications [50, 52]. A recent study in NMOSD reported that the probability of complete recovery decreased from 50% if PE started immediately to 1–5% if it started at day 20, suggesting PE should be started as soon as possible in addition to steroids [51]. Immunoadsorption (IA) is an alternative apheresis therapy to PE when PE is unavailable or contraindicated [53]. Intravenous immunoglobulin (IVIG) therapy is much more widely used and advantageous, because it can be administered without any special requirements [54, 55]. Oral corticosteroids with 3–4 month taper commonly serve as maintenance treatments and a bridge to steroid-sparing maintenance treatments following an NMOSD exacerbation. First-line medications for maintenance treatment in the chronic phase of NMOSD include off label use of azathioprine (AZA), MMF, and rituximab (RTX). Other medications that have also been used less commonly include methotrexate, IVIG, mitoxantrone, and cyclophosphamide [5, 18, 56]. Although there are several newly approved medications in randomization studies such as B cell depleting agents (inebilizumab), anti-interleukin-6 receptor monoclonal antibodies (satralizumab and tocilizumab), and complement blocking agent (eculizumab) for maintenance treatment in NMOSD, long-term benefits, and side effects need evaluation. Because only half of MOG-ON patients will relapse and the recovery from each attack is usually fairly good, long-term therapy is not needed until relapses occur. Studies have found that commonly used immunotherapies (AZA, MMF), IVIG, rituximab, and tocilizumab significantly reduced annualized relapse rate in MOGAD [57–59].

By analyzing publications on ON over the past 10 years, the future challenges and outstanding questions are to clarify mechanisms underlying demyelination, combine novel biomarkers and neuroimaging to improve diagnostic accuracy, promote the development of more effective treatments and develop potential neuroprotective drugs to prevent axonal loss or remyelinating treatments for better visual outcomes and recovery [4, 60, 61].

In this study, publications on ON extracted from the WOS database were analyzed comprehensively and objectively. Being an exploratory study there were some limitations. First, the data were extracted only from the WOS database, which may not reflect the complete set of research in the field. Second, due to the limitations of this database, probable unpublished articles were not included, which may cause publication bias. However, this effect is likely to have been small and not to have substantially impacted the overall results. Third, only high-frequency keywords were selected for our study, but we cannot exclude some low-frequency terms as possible future research hotspots. Fourth, the software cannot distinguish between the relative contributions of multiple authors. Fifth, although objective analysis is available, there may be inherent subjective bias in the interpretation of results due to the lack of uniform standard for setting parameters.

## Conclusions

Substantial developments in ON have occurred over the past decade, and we constructed a series of science maps to depict the research output and knowledge structure. Because research is becoming increasingly collaborative, the research trends and hotspots of this study may also be helpful for neuro-ophthalmologists in choosing appropriate organizations or authors for collaboration.

## Supplementary Information

Below is the link to the electronic supplementary material.

Supplementary file1 (DOCX 23 KB)
