# Supplementary material for: Visualization and analysis of mapping knowledge domains for optic neuritis: a bibliometric research from 2013 to 2022
Source: Int Ophthalmol. 2024 Feb 12;44(1):57. doi: 10.1007/s10792-024-02948-7 (PMC10859331; doi:10.1007/s10792-024-02948-7)
Supplement: Supplementary file 1 — Supplementary file1 (DOCX 23 KB) [file 10792_2024_2948_MOESM1_ESM.docx]

**Supplement 1.** Reference co-citation analysis.

| num. | references | cluster |
| --- | --- | --- |
| 1 | polman ch, 2011, ann neurol, v69, p292, doi 10.1002/ana.22366 | 1 |
| 2 | beck rw, 1992, new engl j med, v326, p581, doi 10.1056/nejm199202273260901 | 1 |
| 3 | thompson aj, 2018, lancet neurol, v17, p162, doi 10.1016/s1474-4422(17)30470-2 | 1 |
| 4 | kurtzke jf, 1983, neurology, v33, p1444, doi 10.1212/wnl.33.11.1444 | 1 |
| 5 | toosy at, 2014, lancet neurol, v13, p83, doi 10.1016/s1474-4422(13)70259-x | 1 |
| 6 | costello f, 2006, ann neurol, v59, p963, doi 10.1002/ana.20851 | 1 |
| 7 | brodsky m, 2008, arch neurol-chicago, v65, p727, doi 10.1001/archneur.65.6.727 | 1 |
| 8 | bech rw, 1991, arch ophthalmol-chic, v109, p1673, doi 10.1001/archopht.1991.01080120057025 | 1 |
| 9 | syc sb, 2012, brain, v135, p521, doi 10.1093/brain/awr264 | 1 |
| 10 | tewarie p, 2012, plos one, v7, doi 10.1371/journal.pone.0034823 | 1 |
| 11 | petzold a, 2010, lancet neurol, v9, p921, doi 10.1016/s1474-4422(10)70168-x | 1 |
| 12 | fisher jb, 2006, ophthalmology, v113, p324, doi 10.1016/j.ophtha.2005.10.040 | 1 |
| 13 | polman ch, 2005, ann neurol, v58, p840, doi 10.1002/ana.20703 | 1 |
| 14 | trip sa, 2005, ann neurol, v58, p383, doi 10.1002/ana.20575 | 1 |
| 15 | petzold a, 2017, lancet neurol, v16, p797, doi 10.1016/s1474-4422(17)30278-8 | 1 |
| 16 | green aj, 2010, brain, v133, p1591, doi 10.1093/brain/awq080 | 1 |
| 17 | petzold a, 2014, nat rev neurol, v10, p447, doi 10.1038/nrneurol.2014.108 | 1 |
| 18 | cruz-herranz a, 2016, neurology, v86, p2303, doi 10.1212/wnl.0000000000002774 | 1 |
| 19 | schippling s, 2015, mult scler j, v21, p163, doi 10.1177/1352458514538110 | 1 |
| 20 | talman ls, 2010, ann neurol, v67, p749, doi 10.1002/ana.22005 | 1 |
| 21 | saidha s, 2015, ann neurol, v78, p801, doi 10.1002/ana.24487 | 1 |
| 22 | walter sd, 2012, ophthalmology, v119, p1250, doi 10.1016/j.ophtha.2011.11.032 | 1 |
| 23 | bennett jl, 2015, mult scler j, v21, p678, doi 10.1177/1352458514567216 | 1 |
| 24 | ratchford jn, 2009, neurology, v73, p302, doi 10.1212/wnl.0b013e3181af78b8 | 1 |
| 25 | balcer lj, 2006, new engl j med, v354, p1273, doi 10.1056/nejmcp053247 | 1 |
| 26 | saidha s, 2011, brain, v134, p518, doi 10.1093/brain/awq346 | 1 |
| 27 | gordon-lipkin e, 2007, neurology, v69, p1603, doi 10.1212/01.wnl.0000295995.46586.ae | 1 |
| 28 | mcdonald wi, 2001, ann neurol, v50, p121, doi 10.1002/ana.1032 | 1 |
| 29 | martinez-lapiscina eh, 2016, lancet neurol, v15, p574, doi 10.1016/s1474-4422(16)00068-5 | 1 |
| 30 | gabilondo i, 2015, ann neurol, v77, p517, doi 10.1002/ana.24351 | 1 |
| 31 | trapp bd, 1998, new engl j med, v338, p278, doi 10.1056/nejm199801293380502 | 1 |
| 32 | compston a, 2008, lancet, v372, p1502, doi 10.1016/s0140-6736(08)61620-7 | 1 |
| 33 | saidha s, 2011, mult scler j, v17, p1449, doi 10.1177/1352458511418630 | 1 |
| 34 | gabilondo i, 2014, ann neurol, v75, p98, doi 10.1002/ana.24030 | 1 |
| 35 | gelfand jm, 2012, brain, v135, p1786, doi 10.1093/brain/aws098 | 1 |
| 36 | naismith rt, 2009, neurology, v72, p1077, doi 10.1212/01.wnl.0000345042.53843.d5 | 1 |
| 37 | parisi v, 1999, invest ophth vis sci, v40, p2520 | 1 |
| 38 | saidha s, 2012, lancet neurol, v11, p963, doi 10.1016/s1474-4422(12)70213-2 | 1 |
| 39 | balcer lj, 2015, brain, v138, p11, doi 10.1093/brain/awu335 | 1 |
| 40 | schneider e, 2013, plos one, v8, doi 10.1371/journal.pone.0066151 | 1 |
| 41 | sepulcre j, 2007, neurology, v68, p1488, doi 10.1212/01.wnl.0000260612.51849.ed | 1 |
| 42 | kupersmith mj, 2002, brain, v125, p812, doi 10.1093/brain/awf087 | 1 |
| 43 | gal rl, 2008, ophthalmology, v115, p1079, doi 10.1016/j.ophtha.2007.08.004 | 1 |
| 44 | hoorbakht h, 2012, open ophthalmol j, v6, p65, doi 10.2174/1874364101206010065 | 1 |
| 45 | saidha s, 2013, jama neurol, v70, p34, doi 10.1001/jamaneurol.2013.573 | 1 |
| 46 | suhs kw, 2012, ann neurol, v72, p199, doi 10.1002/ana.23573 | 1 |
| 47 | klistorner a, 2008, ann neurol, v64, p325, doi 10.1002/ana.21474 | 1 |
| 48 | raftopoulos r, 2016, lancet neurol, v15, p259, doi 10.1016/s1474-4422(16)00004-1 | 1 |
| 49 | ratchford jn, 2013, neurology, v80, p47, doi 10.1212/wnl.0b013e31827b1a1c | 1 |
| 50 | rodriguez m, 1995, neurology, v45, p244, doi 10.1212/wnl.45.2.244 | 1 |
| 51 | sato dk, 2014, neurology, v82, p474, doi 10.1212/wnl.0000000000000101 | 2 |
| 52 | kitley j, 2014, jama neurol, v71, p276, doi 10.1001/jamaneurol.2013.5857 | 2 |
| 53 | jarius s, 2016, j neuroinflamm, v13, doi 10.1186/s12974-016-0717-1 | 2 |
| 54 | jarius s, 2016, j neuroinflamm, v13, doi 10.1186/s12974-016-0718-0 | 2 |
| 55 | ramanathan s, 2018, j neurol neurosur ps, v89, p127, doi 10.1136/jnnp-2017-316880 | 2 |
| 56 | jurynczyk m, 2017, brain, v140, p3128, doi 10.1093/brain/awx276 | 2 |
| 57 | ramanathan s, 2016, mult scler j, v22, p470, doi 10.1177/1352458515593406 | 2 |
| 58 | mader s, 2011, j neuroinflamm, v8, doi 10.1186/1742-2094-8-184 | 2 |
| 59 | kitley j, 2012, neurology, v79, p1273, doi 10.1212/wnl.0b013e31826aac4e | 2 |
| 60 | krupp lb, 2013, mult scler j, v19, p1261, doi 10.1177/1352458513484547 | 2 |
| 61 | cobo-calvo a, 2018, neurology, v90, pe1858, doi 10.1212/wnl.0000000000005560 | 2 |
| 62 | pache f, 2016, j neuroinflamm, v13, doi 10.1186/s12974-016-0720-6 | 2 |
| 63 | chen jj, 2018, am j ophthalmol, v195, p8, doi 10.1016/j.ajo.2018.07.020 | 2 |
| 64 | hoftberger r, 2015, mult scler j, v21, p866, doi 10.1177/1352458514555785 | 2 |
| 65 | ramanathan s, 2014, neurol-neuroimmunol, v1, doi 10.1212/nxi.0000000000000040 | 2 |
| 66 | kim sm, 2015, neurol-neuroimmunol, v2, doi 10.1212/nxi.0000000000000163 | 2 |
| 67 | waters p, 2015, neurol-neuroimmunol, v2, doi 10.1212/nxi.0000000000000089 | 2 |
| 68 | hacohen y, 2018, jama neurol, v75, p478, doi 10.1001/jamaneurol.2017.4601 | 2 |
| 69 | ogawa r, 2017, neurol-neuroimmunol, v4, doi 10.1212/nxi.0000000000000322 | 2 |
| 70 | reindl m, 2013, nat rev neurol, v9, p455, doi 10.1038/nrneurol.2013.118 | 2 |
| 71 | reindl m, 2019, nat rev neurol, v15, p89, doi 10.1038/s41582-018-0112-x | 2 |
| 72 | hennes em, 2017, neurology, v89, p900, doi 10.1212/wnl.0000000000004312 | 2 |
| 73 | akaishi t, 2016, j neurol neurosur ps, v87, p446, doi 10.1136/jnnp-2014-310206 | 2 |
| 74 | rostasy k, 2012, arch neurol-chicago, v69, p752, doi 10.1001/archneurol.2011.2956 | 2 |
| 75 | jarius s, 2018, j neuroinflamm, v15, doi 10.1186/s12974-018-1144-2 | 2 |
| 76 | zamvil ss, 2015, neurol-neuroimmunol, v2, doi 10.1212/nxi.0000000000000062 | 2 |
| 77 | baumann m, 2015, j neurol neurosur ps, v86, p265, doi 10.1136/jnnp-2014-308346 | 2 |
| 78 | jurynczyk m, 2017, brain, v140, p617, doi 10.1093/brain/aww350 | 2 |
| 79 | ramanathan s, 2016, autoimmun rev, v15, p307, doi 10.1016/j.autrev.2015.12.004 | 2 |
| 80 | probstel ak, 2011, neurology, v77, p580, doi 10.1212/wnl.0b013e318228c0b1 | 2 |
| 81 | jarius s, 2018, nervenarzt, v89, p1388, doi 10.1007/s00115-018-0607-0 | 2 |
| 82 | wingerchuk dm, 2015, neurology, v85, p177, doi 10.1212/wnl.0000000000001729 | 3 |
| 83 | lennon va, 2004, lancet, v364, p2106, doi 10.1016/s0140-6736(04)17551-x | 3 |
| 84 | wingerchuk dm, 2006, neurology, v66, p1485, doi 10.1212/01.wnl.0000216139.44259.74 | 3 |
| 85 | wingerchuk dm, 2007, lancet neurol, v6, p805, doi 10.1016/s1474-4422(07)70216-8 | 3 |
| 86 | wingerchuk dm, 1999, neurology, v53, p1107, doi 10.1212/wnl.53.5.1107 | 3 |
| 87 | lennon va, 2005, j exp med, v202, p473, doi 10.1084/jem.20050304 | 3 |
| 88 | jarius s, 2012, j neuroinflamm, v9, doi 10.1186/1742-2094-9-14 | 3 |
| 89 | kitley j, 2012, brain, v135, p1834, doi 10.1093/brain/aws109 | 3 |
| 90 | waters pj, 2012, neurology, v78, p665, doi 10.1212/wnl.0b013e318248dec1 | 3 |
| 91 | kim hj, 2015, neurology, v84, p1165, doi 10.1212/wnl.0000000000001367 | 3 |
| 92 | trebst c, 2014, j neurol, v261, p1, doi 10.1007/s00415-013-7169-7 | 3 |
| 93 | matiello m, 2008, neurology, v70, p2197, doi 10.1212/01.wnl.0000303817.82134.da | 3 |
| 94 | lucchinetti cf, 2002, brain, v125, p1450, doi 10.1093/brain/awf151 | 3 |
| 95 | kleiter i, 2016, ann neurol, v79, p206, doi 10.1002/ana.24554 | 3 |
| 96 | pittock sj, 2006, arch neurol-chicago, v63, p390, doi 10.1001/archneur.63.3.390 | 3 |
| 97 | jarius s, 2010, j neurol sci, v298, p158, doi 10.1016/j.jns.2010.07.011 | 3 |
| 98 | pittock sj, 2006, arch neurol-chicago, v63, p964, doi 10.1001/archneur.63.7.964 | 3 |
| 99 | mealy ma, 2012, arch neurol-chicago, v69, p1176, doi 10.1001/archneurol.2012.314 | 3 |
| 100 | pittock sj, 2008, arch neurol-chicago, v65, p78, doi 10.1001/archneurol.2007.17 | 3 |
| 101 | weinshenker bg, 2006, ann neurol, v59, p566, doi 10.1002/ana.20770 | 3 |
| 102 | papadopoulos mc, 2012, lancet neurol, v11, p535, doi 10.1016/s1474-4422(12)70133-3 | 3 |
| 103 | jiao yj, 2013, neurology, v81, p1197, doi 10.1212/wnl.0b013e3182a6cb5c | 3 |
| 104 | pandit l, 2015, mult scler j, v21, p845, doi 10.1177/1352458515572406 | 3 |
| 105 | takahashi t, 2007, brain, v130, p1235, doi 10.1093/brain/awm062 | 3 |
| 106 | jarius s, 2011, j neurol sci, v306, p82, doi 10.1016/j.jns.2011.03.038 | 3 |
| 107 | jarius s, 2014, clin exp immunol, v176, p149, doi 10.1111/cei.12271 | 3 |
| 108 | kleiter i, 2012, arch neurol-chicago, v69, p239, doi 10.1001/archneurol.2011.216 | 3 |
| 109 | merle h, 2012, arch ophthalmol-chic, v130, p858, doi 10.1001/archophthalmol.2012.1126 | 3 |
| 110 | kidd d, 2003, brain, v126, p276, doi 10.1093/brain/awg045 | 3 |
